# The diagnostic value of Doppler ultrasonography after pediatric kidney transplantation

**DOI:** 10.1007/s00467-021-05253-y

**Published:** 2021-09-03

**Authors:** Doris Franke

**Affiliations:** Pediatric Ultrasonography, Clinic for Pediatric Kidney, Liver and Metabolic Diseases, Hannover Medical School, Carl-Neuberg-Str 1, 30625 Hannover, Germany

**Keywords:** Pediatric kidney transplantation, Vascular complications, Ultrasonography, Doppler, CEUS

## Abstract

Ultrasonography (US) plays a major diagnostic role in the pre- and post-transplant evaluation of recipient and donor. In most cases, US remains the only necessary imaging modality. After pediatric kidney transplantation, US can ensure immediate bedside diagnosis of vessel patency and possible postoperative non-vascular complications. Criteria for US diagnosis of kidney vessel thrombosis and stenosis in the transplant will be presented. Non-vascular complications after kidney transplantation include hydronephrosis, hematoma, lymphocele, and abscess. US can detect suggestive, but nevertheless non-specific, acute signs (sudden increase in volume and elevated resistive index), and chronic rejection, which therefore remains a histological diagnosis. US is of little or no help in detection of tubular necrosis or drug toxicity, but it can exclude other differential diagnoses. This educational review provides a practical and systematic approach to a multimodal US investigation of the kidney transplant. It includes a short overview on possible indications for contrast-enhanced ultrasonography (CEUS) in children after kidney transplantation.

## Introduction

Kidney transplantation is the treatment of choice in children with kidney failure.

Ultrasonography (US) is the first-line imaging modality after kidney transplantation and most often the only one available due to its overall accessibility and the advantage of bedside investigations without need of transportation, sedation, or general anesthesia. After kidney transplantation, complications can be divided into early and late in the clinical course as well as vascular and non-vascular. The latter include parenchymal abnormalities, urological complications of the collecting system, different perinephritic fluid collections, and complications after kidney biopsy (Table 1). This educational review concentrates on Doppler US for mostly vascular complications.

**Table 1:** Possible vascular and non-vascular ultrasonographic findings and complications after pediatric kidney transplantation

Vascular	Non-vascular
Kidney vessel thrombosis	Hydronephrosis
Kidney vein obstruction	Perinephritic fluid collections
Kidney artery stenosis	Urine leakage
Arteriovenous fistula	(seroma, lymphocele, hematoma)
Aneurysm	Free fluid (abdomen, pleural effusion)
Infarction	Unclear focal lesions
	Cysts, calcifications, stones

The avoidance of ionized radiation is of special importance in children, who are more radiosensitive [1–3] and already have an increased overall tumor risk because of chronic kidney failure. Using US and, if indicated, ultrasonography-contrast agents (US-CA), the possible nephrotoxic side effects of radiological contrast agents and nephrogenic systemic fibrosis, or cerebral deposits after gadolinium-based MRI contrast agents, can be avoided [4].

Ultrasound is non-invasive, easily available, can be used bedside, and is cost-effective. Advancements in ultrasound imaging technology and new techniques such as contrast-enhanced ultrasound (CEUS) or modalities such as B-Flow (GE Healthcare, Milwaukee, MI, USA) or Superb Micro-vascular Imaging (SMI, Canon Medical, Tustin, CA, USA) may improve early detection of anatomical or vascular abnormalities.

Detailed information on the history, present clinical status of the patient, transplant particularities, such as multiple vessels or intra-or extraperitoneal position of the graft, and precise questions are essential for the US investigator as the quality of the scan is very much dependent on the knowledge of what, besides the routine scan, should be sought in the US examination.

## Timing of ultrasound imaging after kidney transplantation

In cases of intraoperative problems, e.g., size mismatch with a large graft and a small recipient, the first ultrasound scan should have already been performed in the operating theatre, as the increased intra-abdominal pressure after abdominal closure may lead to an alteration of the kidney position and kinking of its vessels.

In a surgically uneventful transplantation, the first kidney scan is performed in many centers immediately after transfer of the patient to the intensive care unit to exclude early vessel occlusion of the artery and vein using color and pulse-wave (PW) Doppler sonography. This is of major importance to save the graft from a major early complication of early failure due to kidney vein or artery thrombosis.

In patients with a normal baseline US scan, the next routine examination is performed on the second postoperative day and — depending on the patient and the standards of the transplant center — once a week thereafter during hospitalization if the clinical course is uneventful. However, standard operating procedures vary from center to center and from country to country. Unexplained reduction of urine production and elevation in kidney retention parameters are indications for immediate additional US. Because of an increased overall tumor risk in children after kidney transplantation, many centers perform one routine ultrasound examination per year, including for the remnant native kidneys, during long-term surveillance, although the optimal screening frequency is controversial and there is a lack of prospective studies concerning its cost-effectiveness [5, 6].

US is also a useful tool for guidance of interventions such as kidney biopsies, positioning of percutaneous nephrostomy catheters in case of major urinary tract obstruction, or placing of suprapubic catheters.

## The ultrasound investigation: a systematic approach

In infants with tiny iliac veins and too little space in the fossa iliaca, the kidney transplant is usually anastomosed to the recipient’s aorta and inferior vena cava. With this approach, an intra-abdominal positioning of the kidney transplant is necessary, which may obscure the scanning window by superimposed intestine. A scanning view from dorsolateral and low frequency probes may overcome this obstacle.

In older children, the anastomoses are performed with the iliac vessels and the kidney is placed in the right or left fossa iliaca extraperitoneally close to the ventral surface. This position ensures excellent conditions for US. The child is in a supine position. Usually, an abdominal convex probe is used for B-Mode US and Doppler measurements. Linear probes with a high frequency and better resolution (5–10 MHz) are used thereafter for better depiction of small structures or subcapsular vessels. A systematic stepwise technical approach should always be taken, starting with:

1. *Grayscale (B-Mode) US*. This allows assessment of organ size, echogenicity, corticomedullary differentiation of the kidney parenchyma, and assessment of perirenal spaces.
2. *Duplex US*: *Color Doppler and PW Doppler.* Vascular patency of the kidney transplant artery and vein, the parenchymal perfusion, and the recipient’s anastomosing vessels are investigated using Duplex sonography. The color-coded Doppler visualizes the vessels and depicts the flow direction (red: blood flow toward the probe, blue: blood flow away from the probe). The PW Doppler depicts the spectral waveform and allows measurements of the flow velocity, acceleration time, and indices, such as the resistive index (RI) [7, 8].
3. The “power Doppler*”* is more sensitive for lower-flow velocities and may help to detect non-perfused areas after kidney infarction.

More recently, some ultrasound systems provide non-Doppler-based flow detection techniques such as B-Flow or SMI using the subtraction method to detect flow in very small vessels with a high spatial resolution and a good distinction of vessels in close proximity [9].

The systematic approach of scanning a patient has always been to include the whole urinary tract:

1. Bladder: the bladder should be empty while a transurethral (or in individual cases suprapubic) urinary catheter is present to ensure healing of the neoureterostomy in the first 5–7 post-operative days. The end of a possible trans-ureteral splint should be seen in the bladder, which is used by many surgeons for initial post-operative splinting of the transplant ureter. Possible hematomas within the bladder can be ruled out. The residual bladder volume after voiding can be estimated by the same ellipsoid formula that is used for the kidney volume.
2. Ureter: the transplant ureter (and the native ureters or a residuum after nephrectomy) should be checked for dilatation, floating particles, splints, or stones.
3. Kidney transplant: size, kidney vessels as far as to the anastomoses, kidney parenchyma, the collecting system, proximal ureter, and the perirenal space are examined. The volume of the transplant kidney can be estimated by the formula: length × depth × width × 0.523.
4. Fluid collections in the pleural and abdominal spaces can be visualized in case of possible overhydration.

## Duplex sonography

Technique.

Using the color-coded Doppler sonography, the kidney artery and vein can be distinguished by the flow direction (artery usually in red — flow toward the probe; vein usually in blue — flow away from the probe). PW measurements should be performed in three different segmental arteries in the upper and lower pole as well as in the middle part of the kidney and in three interlobular arteries in their course beneath the medullae. Normal values of the flow velocities and RI in school-age children are as follows: Kidney artery V systolic 80.0 ± 18.0 cm/s, V diastolic 34.2 ± 9.6 cm/s, RI 0.71 ± 0.09; Segmental artery V systolic 45.5 ± 9.1 cm/s, V diastolic 15.5 ± 4.5 cm/s, RI 0.66 ± 0.08; Interlobar artery V systolic 27.9 ± 5.3 cm/s, V diastolic 11.3 ± 2.7 cm/s, RI 0.58 ± 0.1 [10]. Measurements of arcuate arteries at the distal end of the medullae are not obligatory.

**Fig. 1:**
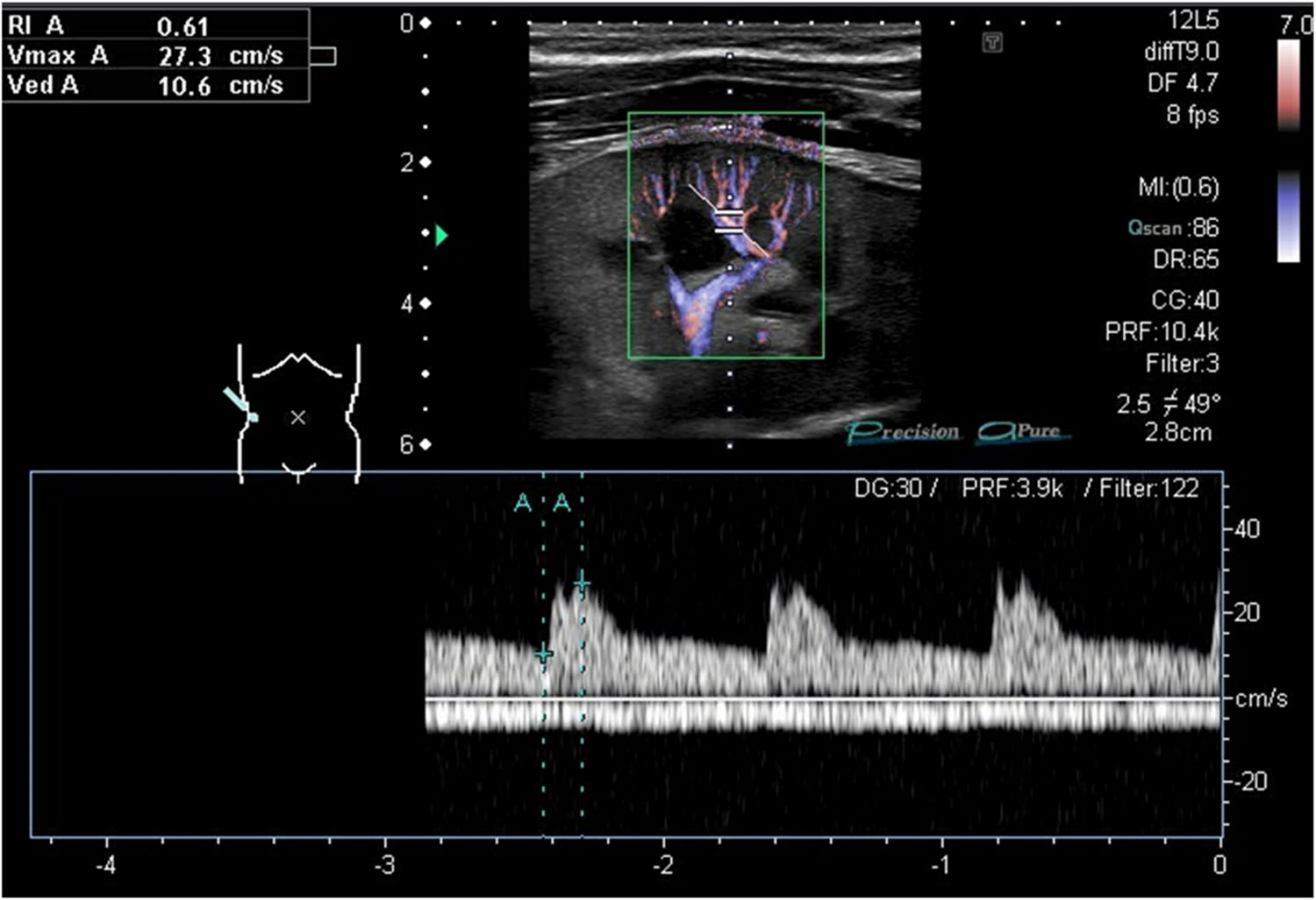
Doppler sonography of an interlobular kidney artery using a bidirectional power Doppler. Signals above the Zero-Line in the pulse-wave (PW)-Doppler indicate a flow toward the probe (interlobular artery), and signals below the Zero-Line indicate a flow away from the probe (kidney vein)

The kidney allograft shows the characteristics of a low-impedance capillary bed with a continuous flow throughout the cardiac cycle. In the PW Doppler spectrum, a rapid systolic rise in the systole is often accompanied by an early systolic peak and a slowly declining continuous diastolic flow (Fig. 1).

The early peak systolic and the end-diastolic flow velocity should be measured. From this, the RI (also referred to as the Pourcelot Index) is calculated according to the formula: peak systolic velocity – end-diastolic velocity/peak systolic velocity.

The RI is independent of the angle and is not a specific, but it is, nevertheless, an important diagnostic parameter. The RI is a measure for pulsatile blood flow and indirectly gives information regarding vessel resistance distally of the measurement position [7] and also of the elasticity of the greater vessels. It may be altered by many factors such as age, heart rate, and the area sampled [8, 11]. The RI values are higher with increasing age in adults and are higher in the hilar region compared to the segmental and interlobular arteries. An isolated elevated RI in a single kidney vessel is of limited value. However, elevated RIs > 0.8 are found in acute and chronic rejection and may indicate delayed graft function and poor graft outcome [12]. In adults, the association of an elevated RI with diabetic nephropathy and glomerulosclerosis has been shown [13]. Other studies suggest that the RI in the transplanted kidney is related to the recipient’s vascular status and not representative for the transplanted kidney itself [14–16]. Reference values for the RI are age-dependent in children and adults in native kidneys [11, 16–19]. Normal values in kidney transplants with stable function are 0.6–0.7 [13]. As a cut-off, the RI should be lower than 0.8 [20]. There is disagreement as to whether the RI can predict graft outcome. Radermacher [20] found a more rapid deterioration of graft function in a large but heterogenous group of patients with an RI > 0.8 (6.6 ± 5.5 years after kidney transplantation) compared to patients with an RI < 0.8 (4.6 ± 4.6 years after kidney transplantation). In this study, elevated RI was also found to be a prognostic marker for patient survival in adults. Mwipatayi et al. found that immediate RI measurements within 24 h after transplantation in patients with an RI > 0.8 were a strong predictor for both delayed graft function and transplant failure [12]. Other authors did not find a correlation of RI and graft outcome in the early post-transplant period within 6 to 12 months [21–23].

There is evidence that the measuring of kidney RI assesses not only the vessel situation in the allograft but also — indirectly — the elasticity in the recipient’s large upstream vessels [14, 15, 18, 19, 24]. This may explain why kidney RI was not found to be a strong predictor for acute rejection or even graft survival in childhood, although data is much scarcer than in adulthood kidney transplantation.

Measurements of the pulsation index have no additive value to those of the RI. The maximal systolic acceleration time of the intraparenchymal arteries is defined as time in seconds to reach the peak systolic velocity in the intraparenchymal arteries irrespective of the waveform [25].

Different Doppler US signs and possible causes are listed in Table 2.

**Table 2:** Doppler imaging pathologies and possible clinical causes after kidney transplantation

Doppler sign	Possible causes
Resistive index (RI) elevated	Increased vascular resistance, e.g., rejection, kidney vein thrombosis, acute tubular necrosis (ATN)
RI low	Poststenotic tardus-parvus pattern High diastolic flow due to low peripheral resistance, e.g., fistula
Aliasing	Arteriovenous fistula, stenosis
Missing vascular signal in a vessel	Thrombosis, infarction
Negative diastolic flow in the kidney artery	Severe rejection, kidney vein thrombosis, leakage of the aortic “Windkessel”, e.g., relevant ductus arteriosus Botalli
Decreased overall vascularity	Chronic or acute kidney transplant failure, rejection, ATN
Decreased focal vascularity	Pyelonephritis with focal interstitial edema, abscess

## Kidney artery thrombosis

Kidney artery thrombosis is a severe complication and occurs most often in very small-sized donor or recipient vessels. The rate of vascular thrombosis is reported to be 2–12% in the general kidney transplant population, including adult recipients [26, 27]. Risk factors are multiple arteries, kinking, hypotension, hypercoagulability states, and predisposing underlying diseases such as congenital nephrotic syndrome, systemic lupus erythematosus, or antiphospholipid antibody syndrome. In B-Mode, echogenic material may be seen within the vessel. There is an absence of severely diminished or reverse diastolic flow in the kidney artery and therefore also no venous flow with Doppler US (color and PW). For increased certainty, the PW with high sensitivity (low pulse repetition frequency) will reveal no parenchymal flow in the small vessels.

## Kidney vein stenosis and thrombosis

**Fig. 2:**
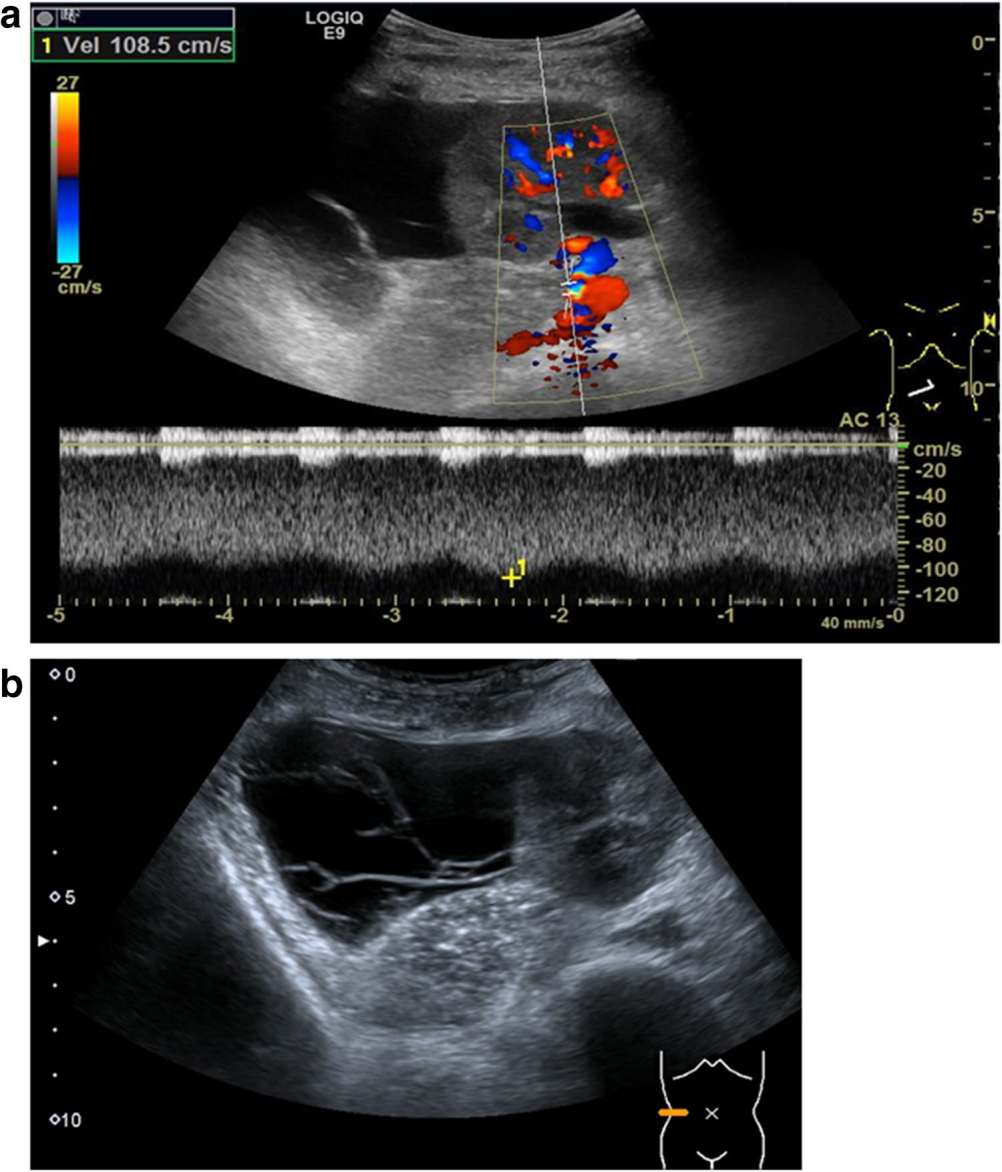
**a**, **b** Kidney vein stenosis due to a lymphocele with kinking of the transplant vessels. **a** Aliasing and highly increased venous velocity of > 100 cm/s in the transplant vein. **b** Lymphocele: echofree fluid collection laterally to the kidney transplant with fibrous septae

Kidney vein stenosis may occur due to tight suturing at the anastomosis or by compression due to kinking or extrarenal fluid collections (Fig. 2b). In B-Mode US, a narrowing of the vessel diameter can be seen with a possible dilatation proximally to the stenosis. Color Doppler US reveals an aliasing artifact and the PW Doppler an increase in the flow velocity (Fig. 2a, b). Kidney vein thrombosis occurs in the early post-transplant phase due to surgical complications, multiple vessels, disparity in vessel size of donor and recipient, prolonged ischemia, hypercoagulable states, hypovolemia, venous compression due to fluid collections such as hematomas or lymphoceles, and severe acute rejection. The reported prevalence is 0.1–4.2% [28].

In grayscale ultrasound, an increase in kidney volume and — possibly — echogenic material within the kidney vein may be seen. If the kidney vein thrombosis persists, the kidney becomes echogenic; in the early phase, it is larger but may later shrink.

Doppler signs include an absent flow in the kidney vein, an abnormal wave pattern in the kidney artery and segmental arteries with reduced velocity, and missing or a reverse diastolic flow. In partial thrombosis, a high RI (> 0.8) can be noted.

## Kidney artery stenosis after kidney transplantation in children

Post-transplantation arterial hypertension in children is common and due to several underlying factors, such as primary kidney disease, side effects of immunosuppressive medication, hormonal disturbances, familial disposition, and kidney artery stenosis.

The diagnosis of kidney artery stenosis is important because it is a correctable form of kidney hypertension and its prevalence after kidney transplantation is estimated to be between 5 and 30% depending on definition of hemodynamic significance and different diagnostic modalities.

Potential risk factors for kidney artery stenosis after transplantation are surgical complications during the process of kidney explantation and transplantation such as vessel damage, intimal dissection, or improper suturing. Furthermore, arteriosclerotic plaques in the donor organ, cytomegalovirus infections, and delayed graft function have been found to be associated with a higher risk.

**Fig. 3:**
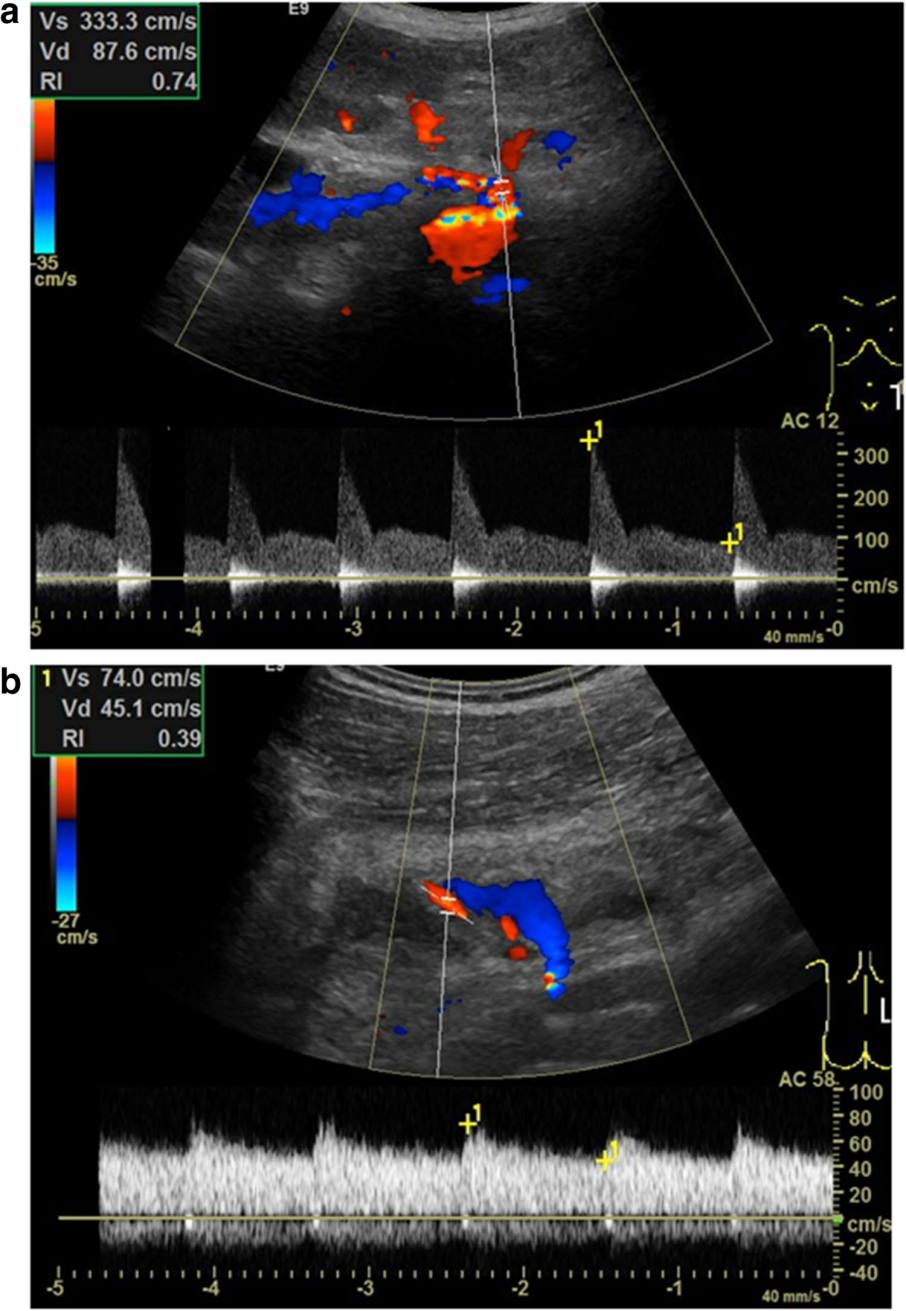
**a**, **b** Kidney artery stenosis of a kidney graft. **a** The maximum systolic velocity is increased to 333 cm/s, aliasing in the kidney artery. **b** Tardus-parvus-pulse in the post-stenotic course indicated by a high diastolic flow and a resulting low resistive index (RI)

In B-Mode US, the diameter and possible narrowing of the kidney artery (> 60%) at the stenosis site should be checked. Color Doppler findings in kidney artery stenosis include an aliasing artifact at the stenosis. In PW Doppler turbulence, spectral broadening (defined as complete filling of the spectral window indicating turbulent flow), a peak systolic velocity > 200 cm/s (cut-off 180–400 cm/s, as suggested in the literature), a poststenotic tardus-parvus-waveform with a prolonged acceleration time (> 0.1 s), a loss of early systolic peak, and a high diastolic flow velocity and low RI are diagnostic (Fig. 3a, b) [25].

## Arteriovenous fistulas and pseudoaneurysms

**Fig. 4:**
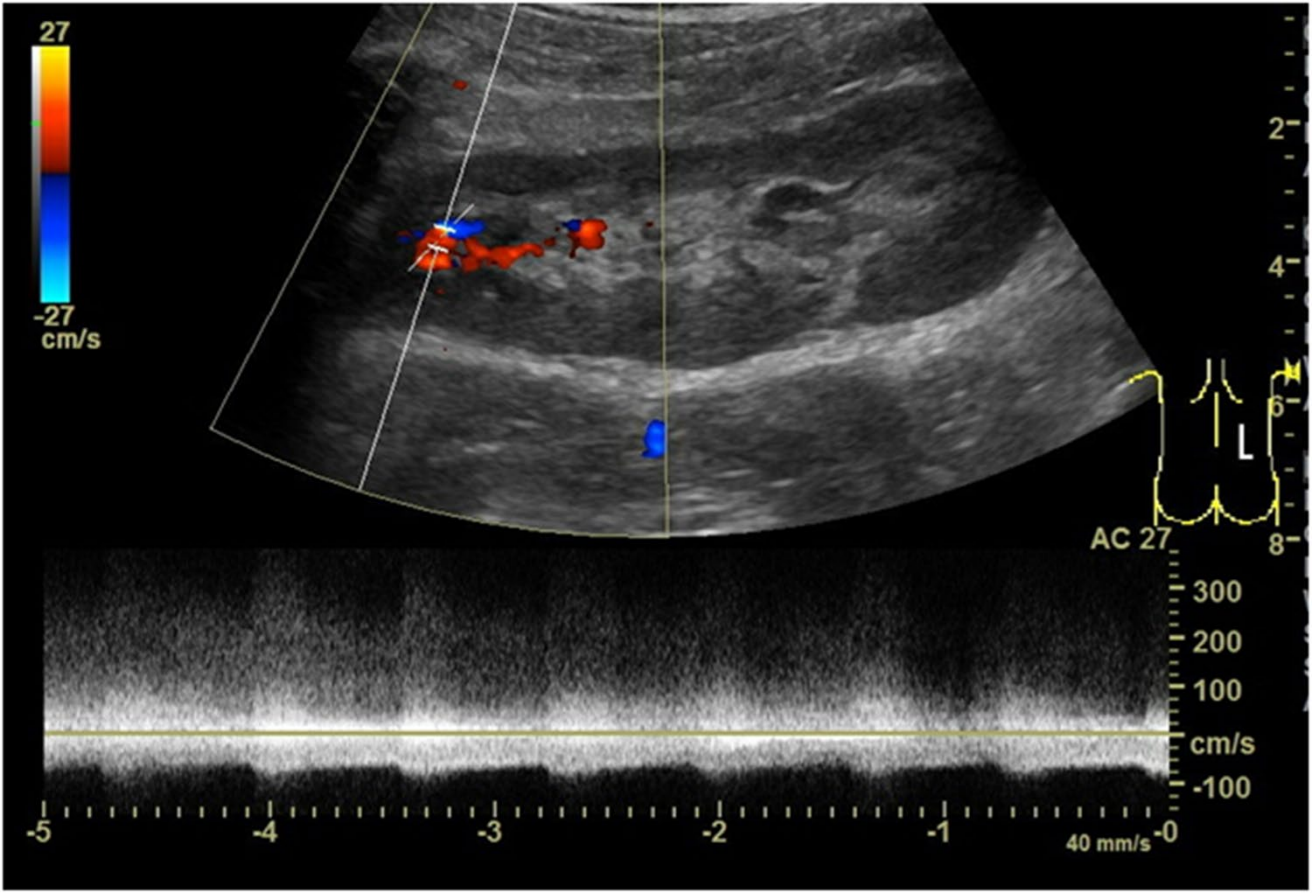
Arteriovenous fistula after kidney biopsy. In the pulse-wave (PW)-Doppler, a turbulent flow pattern with a high flow velocity of > 300 cm/s is depicted

Arteriovenous fistulas and pseudoaneurysms are most often iatrogenic after kidney biopsies and usually resolve spontaneously. Arteriovenous fistulas are only occasionally responsible for a steal phenomenon. In grayscale ultrasound, the feeding vessel may already be seen within the transplant. Using duplex sonography, a turbulent flow with an aliasing artifact, a high flow velocity within the feeding artery and the draining vein, and a low RI due to a high diastolic flow can be detected (Fig. 4). Pseudoaneurysms result from injury of the vessel wall during biopsy, trauma, or after infection. In grayscale ultrasound, they appear as a cyst-like structure due to the development of a pseudoaneurysm with intense bidirectional flow on color imaging.

## Segmental infarction

Segmental infarction may result after ligation or thrombosis of a pole or segmental artery and for thromboembolic, infectious, or inflammatory reasons. Because of angle problems when scanning the lower and upper pole, segmental infarction with lack of detectable vessels in the ischemic lesion may be difficult to diagnose in Doppler or even power Doppler sonography; here CEUS is much more sensitive and has no nephrotoxic side effects. In grayscale US, no abnormalities are seen in the very early phase, but hours later, a typically triangular echopoor area appears.

## Parenchymal abnormalities

**Fig. 5:**
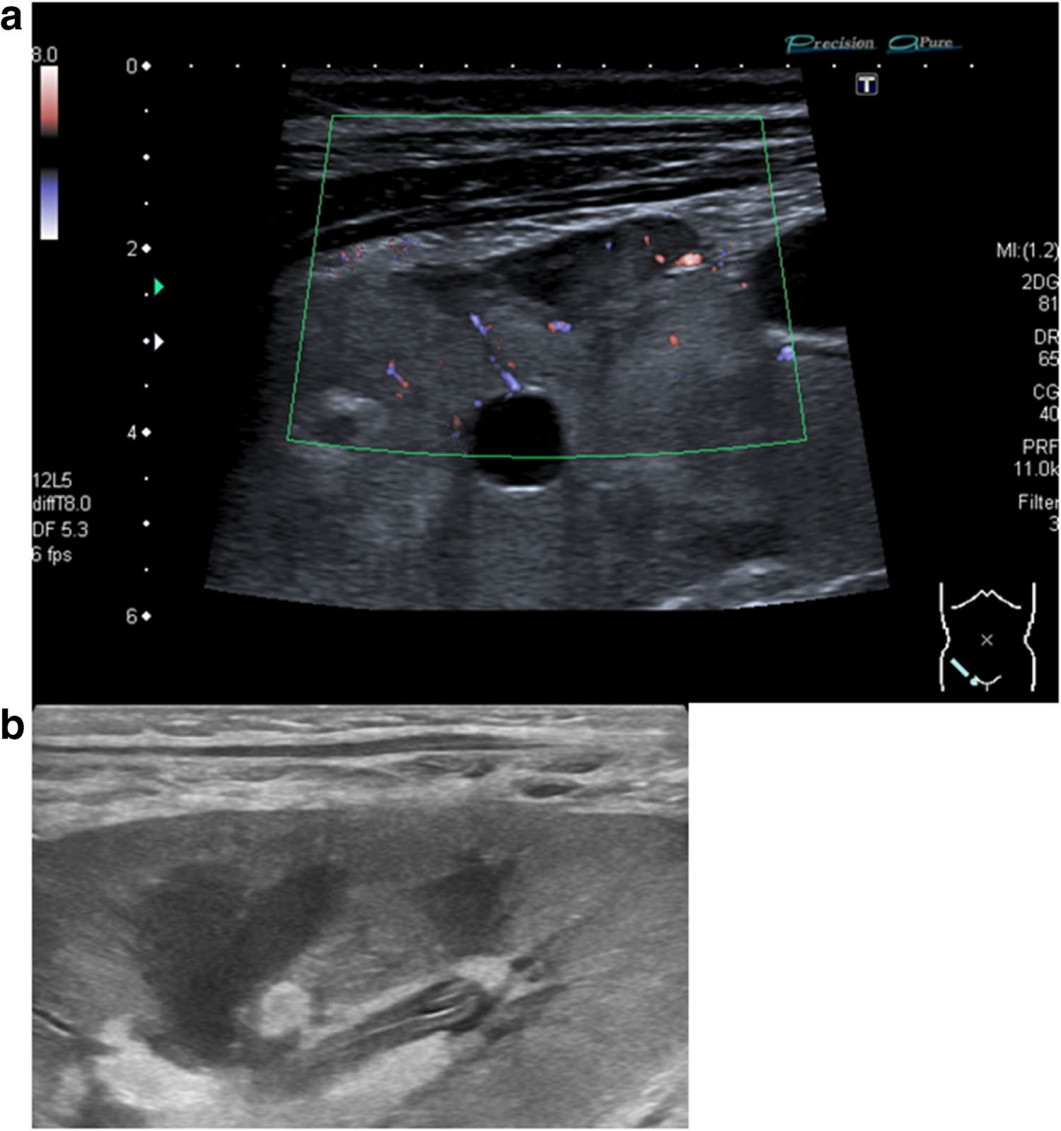
**a** Kidney abscess after transplant urosepsis. **b** Urosepsis, positive urothelial sign

There are no specific US signs in diffuse parenchymal disorders such as acute tubular necrosis, drug toxicity, or early mild rejection. Focal processes such as cysts, tumors, and abscesses (Fig. 5) can be differentiated.

## Perirenal and other fluid collections

Perirenal and other fluid collections should be sought at each US examination.

**Fig. 6:**
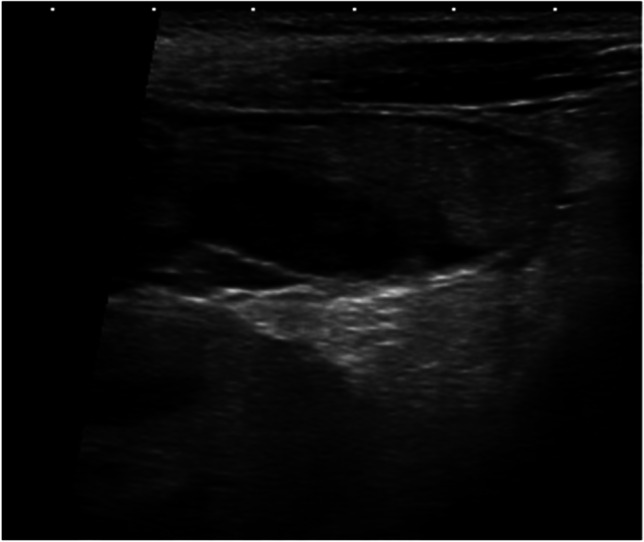
Hematoma after kidney biopsy. Longitudinal section through the kidney transplant with a mixed echorich-echopoor oval mass on top. Linear probe

After acute bleeding, hematomas are echofree and become echogenic after coagulation. In the phase of reorganization and resolution, they change to mixed echorich-echopoor (Fig. 6). No Doppler signals can be visualized within the hematoma. Origins may be intraoperative, after acute bleeding and after kidney biopsy.

Using US, a seroma cannot be confidently distinguished from a urinoma after urinary leakage.

Lymphoceles usually appear from the second week after transplantation onwards, as new echofree, painless fluid collections around the transplant due to lymphatic vessel stripping during the explantation of the graft (Fig. 2a, b). Other fluid collections may be found as free fluid in the abdomen or pleural spaces in situations of overhydration.

## Hyperacute, acute, and chronic rejection

**Fig. 7:**
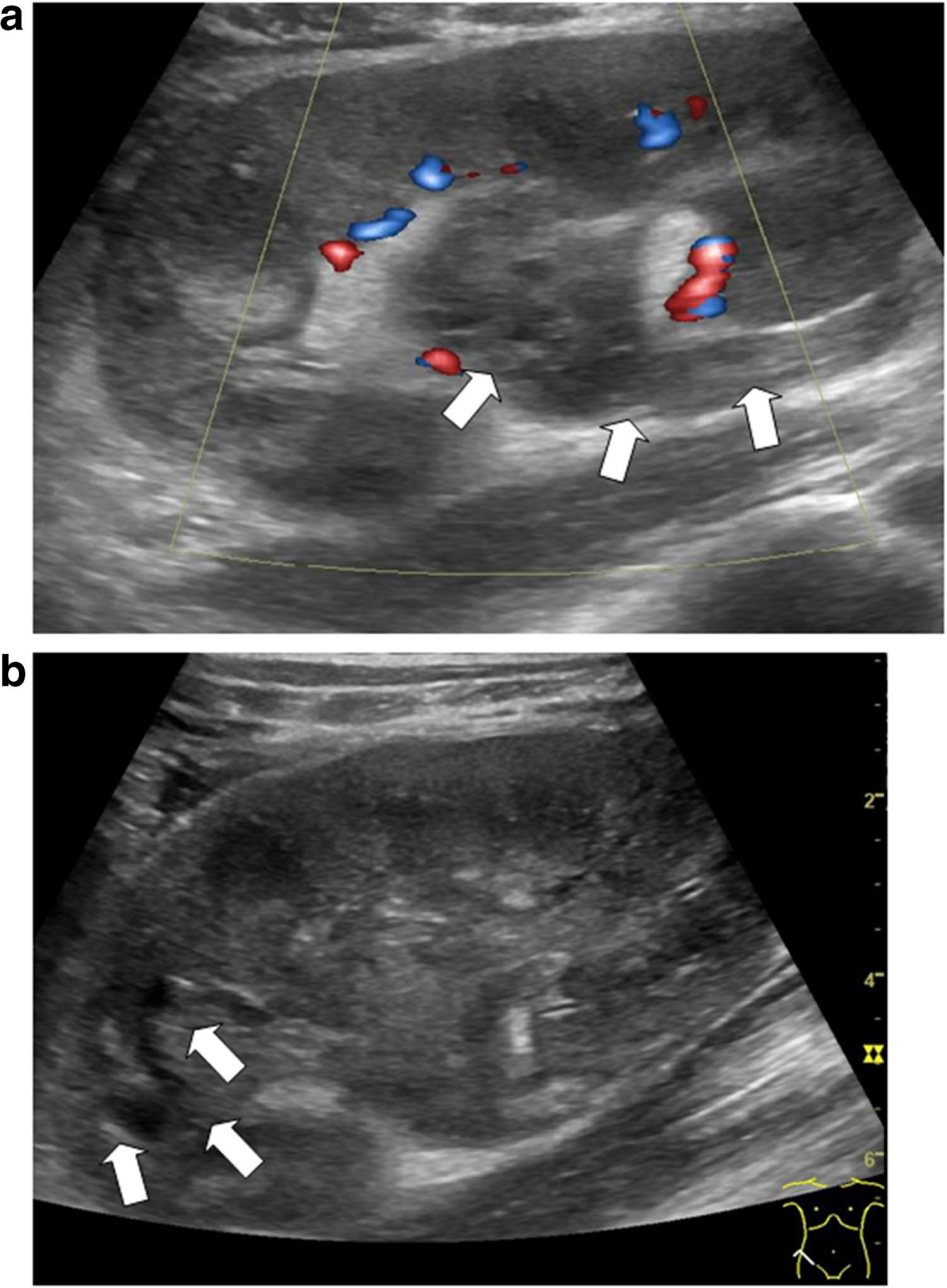
**a** Severe acute rejection with macrohematuria because of inner bleeding/rupture into the pelvis in a 10-year-old girl with chronic graft nephropathy. The girl had already returned to hemodialysis after recurrent antibody-mediated rejection episodes. On palpation “hard” kidney. Nephrectomy and histologically acute and chronic antibody mediated rejection with macroscopic blood clots. Power Doppler with overall reduced vascularity, inhomogeneous material (blood/hematoma) in the renal pelvis and proximal ureter (arrows), longitudinal section. **b** Same 10-year-old patient, longitudinal section. Massive organ swelling, echopoor line in the upper kidney transplant pole indicating inner rupture and bleeding (arrows), proven pathologically after transplant nephrectomy

Rejection may be hyperacute, acute, or chronic depending on the time interval after kidney transplantation. *Hyperacute rejection* is recognized intraoperatively; the reasons are preformed antibodies. The incidence of *acute rejection* has reduced substantially over the past decades due to improved immunosuppressive regimens. Ultrasonographic signs are an interindividual increase of kidney transplant volume compared to baseline measurements. In Doppler US, RIs are elevated > 0.8 due to impaired diastolic flow as a result of organ swelling. In very severe cases, the diastolic flow may even be reversed. A patent kidney vein excludes renal venous thrombosis as a potential differential diagnosis. In hyperacute or acute rejection, massive organ swelling, rupture, and bleeding may be observed as potentially life-threatening complications (Fig. 7a, b).

**Fig. 8:**
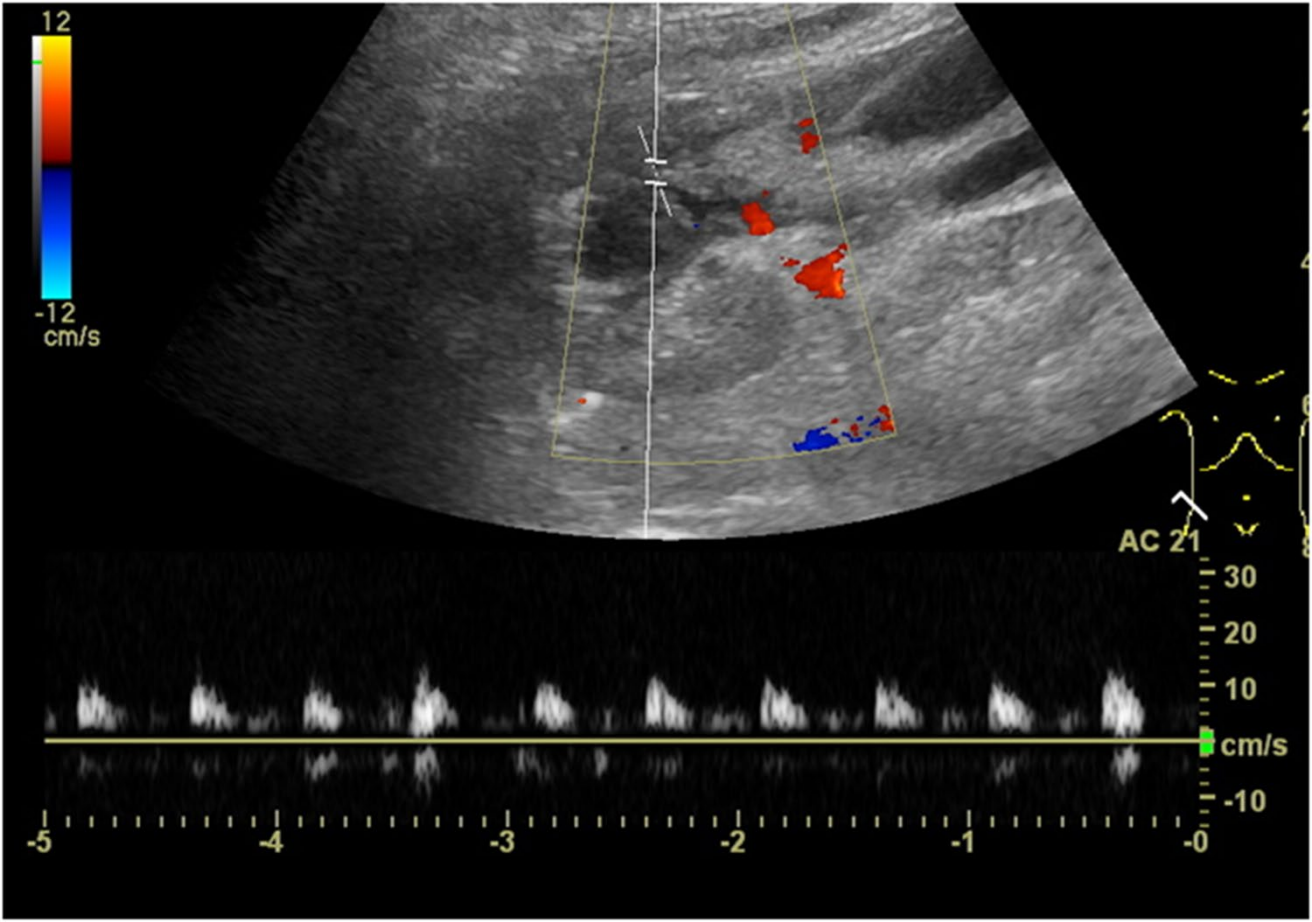
Chronic allograft nephropathy: echogenic kidney with reduced corticomedullary differentiation and scarce vascularity. In the pulse-wave (PW)-Doppler broad systolic peaks, low flow velocities (< 15 cm/s) and reduced-absent end diastolic flow

*Chronic rejection* with histologically interstitial fibrosis and sclerosing vasculitis may present in B-Mode US with an increased organ echogenicity, reduced corticomedullary differentiation, and cortical thinning [29]. Doppler US may reveal reduced overall vascularity and diminished flow velocities (Fig. 8).

## Possible indications for CEUS after kidney transplantation

In Europe, the intravenous application of the second-generation US-CA (SonoVue®, Bracco, Milan, Italy) in children and adolescents up to the age of 18 years is off-label for all indications. In 2016, the FDA granted approval in the USA for Lumasone® (same substance, different name) for liver indications and voiding urosonography, but in Europe, only SonoVue® for voiding urosonography has been authorized (in 2017). Until now, the most frequent indication for CEUS is diagnosis or exclusion of vesicoureteral reflux [30, 31]. However, all intravenous CEUS examinations of kidney transplants in children are off-label.

Advantages of CEUS compared to MRI or CT are the general availability of US, possibility of mobile investigations, lower costs, and the lack of general anesthesia even in infants.

The most important advantage of CEUS in children is the potential for reducing the amount of ionizing radiation by lowering the frequency of CT and radiological scanning. Furthermore, US-CA are eliminated from the body minutes after the examination without being nephro-, cardio-, or hepatotoxic and without known organ deposition, which may be another advantage compared to the unknown effects of detectable gadolinium storage in the brain after repeated examinations despite normal kidney function [32, 33]. The use of US-CA in childhood is effective and safe [34–37].

## Potential indications for intravenous use of CEUS in children

Possible indications for off-label intravenous CEUS of the transplant kidney in childhood are detection of abscesses after transplant, pyelonephritis, unclear focal organ lesions, perfusion disturbances such as vessel patency and infarction, and differentiating of complicated cysts.

## Sonoelastography

Sonoelastography measures the stiffness of the kidney parenchyma. The possible value could be the noninvasive assessment of the degree of organ fibrosis using shearwave or ARFI sonoelastography. In adults, there are conflicting data comparing sonoelastography with histopathological changes in kidney biopsies. There are currently few data from children and, as yet, no multicenter studies with higher patient numbers [38, 39].

## Conclusions

Overall, US is an available and immediate diagnostic tool for the pre- and post-transplantation surveillance of children with a kidney transplant. Major vascular complications such as kidney vessel stenosis or thrombosis can be diagnosed early and with safety. Non-vascular complications such as hydronephrosis, hematoma and lymphocele can be easily assessed.

Kidney US is not a substitute for kidney biopsy in cases of questionable rejection. The rapid development of new US techniques is promising for further advances in the future.

## Key summary points

- Ultrasonography (US) is the first-line imaging tool in the pre- and post-transplant evaluation of a kidney transplant recipient and donor.
- US can ensure immediate bedside diagnosis of vascular complications after kidney transplantation such as stenosis, thrombosis, aneurysm, or infarction as well as possible non-vascular complications (hydronephrosis, hematoma, lymphocele, urinary leakage, and abscess).
- In acute rejection, US can detect only non-specific signs (sudden increase in volume and elevated resistive index) and exclude differential diagnoses.
- In detection of tubular necrosis or drug toxicity, US is of little or no help apart from exclusion of possible differential diagnoses.

## Multiple choice questions (answers are provided following the reference list)

1. Which answer is wrong? Complications after kidney transplantation accurately detected by Ultrasonography are:
  - Hydronephrosis.
  - Tubular necrosis.
  - Kidney artery stenosis.
  - Kidney vein thrombosis.
2. Ultrasonographic signs of a kidney vein thrombosis may be:
  - Nondetection of the kidney vein by Doppler US.
  - High RI in the kidney artery.
  - Missing end diastolic flow in the kidney arteries.
  - All of the above.
3. Which of the following statements on the value of US in acute kidney graft rejection is not correct?
  - Interindividual resistive indices (RIs) can be higher than before the rejection.
  - A sudden increase in the total kidney volume may indicate acute rejection.
  - US can be substituted for kidney graft biopsy in diagnosing acute rejection.
  - RIs above 0.8 can be suggestive of acute rejection.
4. Which of the following statements on arteriovenous fistulas in the kidney graft is correct:
  - Arteriovenous fistulas usually resolve spontaneously.
  - The most common reason for arteriovenous fistulas is a kidney biopsy.
  - A steal phenomenon may result from large arteriovenous fistulas.
  - All of the above.
5. Which of the following statements on the diagnostic value of US is not correct?
  - Seromas can be distinguished from ureteral leakage by US.
  - Kidney artery stenosis can be excluded by US.
  - Segmental renal infarction may be seen by power Doppler US.
  - The sudden increase of intraindividual kidney volume can be suggestive of acute rejection.

## Abbreviations

CEUS: Contrast-enhanced ultrasonography
PW: Pulse-wave Doppler
RI: Resistive index
SMI: Superb micro-vascular imaging
US: Ultrasonography
US-CA: Ultrasonography-contrast agents
